# Effects of 1,25(OH)_2_D_3_ and vitamin D receptor on peripheral CD4^+^/CD8^+^ double‐positive T lymphocytes in a mouse model of systemic lupus erythematosus

**DOI:** 10.1111/jcmm.13037

**Published:** 2017-01-07

**Authors:** Yan Ding, Wang Liao, Xiao‐Jie He, Wei Xiang

**Affiliations:** ^1^ Department of DermatologyMaternal and Child Health Care Hospital of Hainan ProvinceHaikouChina; ^2^ Department of CardiologyHainan General HospitalHaikouChina; ^3^ Department of PediatricsThe Second Xiangya HospitalCentral South UniversityChangshaChina; ^4^ Laboratory of Pediatric NephrologyInstitute of PediatricsCentral South UniversityChangshaChina; ^5^ Department of PediatricsMaternal and Child Health Care Hospital of Hainan ProvinceHaikouChina

**Keywords:** 1,25(OH)_2_D_3_, vitamin D receptor, systemic lupus erythematosus, CD4^+^, CD8^+^, T lymphocytes, mouse model

## Abstract

This study aims to explore effects of 1,25(OH)_2_D_3_ and vitamin D receptor (VDR) on peripheral CD4^+^/CD8^+^ double‐positive (DP) T lymphocytes in systemic lupus erythematosus (SLE). MRL‐LPr/LPr mice with SLE (*n* = 20) and normal MRL mice (*n* = 20) were assigned into the control group (normal mice, without feeding with 1,25(OH)_2_D_3_), the 1,25(OH)_2_D_3_ group (SLE mice, feeding with 1,25(OH)_2_D_3_), the VDR‐knock‐in + 1,25(OH)_2_D_3_ group (SLE mice, VDR‐knock‐in, feeding with 1,25(OH)_2_D_3_) and the VDR‐knockout group (normal mice, VDR‐knockout, without feeding with 1,25(OH)_2_D_3_) (*n* = 10 per group). Levels of T lymphocytes were measured by flow cytometry. The mRNA and proteins expressions of inflammatory factors were measured by qRT‐PCR and ELISA. Extracellular signal‐regulated kinase‐1/2 (ERK1/2) expression was measured by Western blotting. Compared with normal mice, SLE mice showed reduced levels of CD4^+^, CD4^+^/CD8^+^ ratio, and DP lymphocytes. The levels of SLE‐related indicators all increased significantly, followed with severe skin ulcers and urinary system infection. With the increase in time, skin ulcers and urinary system infection were significantly improved, levels of CD4^+^, CD4^+^/CD8^+^ ratio, and DP lymphocytes increased, and levels of SLE‐related indicators all decreased in the 1,25(OH)_2_D_3_ group. There were no significant changes in bioindicators in the control and the VDR‐knock‐in + 1,25(OH)_2_D_3_ groups. The symptoms of SLE gradually occurred in the VDR‐knockout group. This study demonstrates that VDR and 1,25(OH)_2_D_3_ could elevate CD4^+^/CD8^+^ DP T lymphocytes and reduce expressions of inflammatory factors, thus inhibiting the development and progression of SLE.

## Introduction

Systemic lupus erythematosus (SLE) is an autoimmune disorder of connective tissues affecting multiple systems and is associated with a variety of clinical manifestations 1. It is estimated that the incidence of SLE in Western Europe is 2.2 to 5 per 100,000 people, and the prevalence rate is 20.5 to 91 per 100,000 people 2. Symptoms of SLE include neurologic complications, hair loss, ulcers, intermittent fevers, rash and arthritis 3. However, majority of SLE patients have no obvious etiological reasons, and the long‐term fluctuations of the disease cannot be fully explained, which may involve genetic (risk alleles), environmental, drug (anti‐allergic drugs) or viral factors (retrovirus) 4, 5. At present, a standard therapy of SLE includes using immunosuppressive drugs to suppress the immune response and excessive inflammation, among which the vitamin D (VD) is an ideal regulator of immune responses 6.

The most active vitamin D metabolite is 1,25(OH)_2_D_3_, a steroid hormone whose synthesis involves the catalytic 1α‐hydroxylase that transforms 25(OH)D_3_ to 1,25 (OH)_2_D_3_, a key factor in maintaining autoimmune tolerance, and participates in a variety of cell functions including differentiation, proliferation and apoptosis 7. The VD insufficiency is associated with the pathogenesis of many autoimmune diseases including SLE, and it has been shown that 1,25 (OH)_2_D_3_ inhibited the maturation of dendritic cells (DCs) and the activation of Th1 cells in SLE patients 8. Vitamin D receptor (VDR), a member of the nuclear receptor superfamily, is also a DNA ligation and transcription factor, which is considered as a chromatin protein connecting 1,25(OH)_2_D_3_ and mediating its biological functions 9. VDR is expressed in all types of immune cells while VD also exerts its functions through the homeostasis of VDR in the immune system, and despite the controversy, it is believed that the *VDR* gene polymorphism is associated with clinical manifestations of SLE 10. Therefore, this paper investigates the effects of 1,25(OH)_2_D_3_ and VDR on the peripheral CD4^+^CD8^+^ DP T lymphocytes in a mouse model of SLE, in order to determine whether 1,25(OH)_2_D_3_ and VDR are involved in the pathogenesis of SLE.

## Materials and methods

### Ethical statement

Animal experiments were designed with the consent of the animal ethics committee, and all the studies were conducted strictly in accordance with international standards 11.

### Model establishment and grouping

Six‐ to eight‐week‐old MRL‐LPr/LPr mice with SLE and normal MRL mice (SLC Inc., Sizuoka, Japan) were selected. There were 20 mice for each type, with half females and half males, weighing 20–24 g. All mice were housed in clean animal rooms at 22–25°C, with normal circadian rhythm. And all mice were given free access to food and drinking water. SLE VDR‐knock‐in mice and normal VDR‐knockout mice models were established and identified by Seajet Scientific Inc. (Beijing, China). The grouping of mice is shown in Table 1, with 10 mice in each group. Mice in the 1,25(OH)_2_D_3_ and the VDR‐knock‐in + 1,25(OH)_2_D_3_ groups were fed with 5 μg/kg of 1,25(OH)_2_D_3_ (the active form of vitamin D_3_, Sigma‐Aldrich, St. Louis, MO, USA) daily by gastric tubes. The mice in the control and the VDR‐knockout groups were fed normally. All mice were fed for 8, 16 and 24 consecutive weeks.

**Table 1:** Grouping of experimental mice

Group	Treatment
1,25(OH)_2_D_3_ group	MRL‐LPr/LPr mice, feeding with 1,25(OH)_2_D_3_
VDR‐knock‐in + 1,25(OH)_2_D_3_ group	MRL‐LPr/LPr mice, VDR‐knock‐in, feeding with 1,25(OH)_2_D_3_
Control group	Normal mice, feeding without 1,25(OH)_2_D_3_
VDR‐knockout group	Normal mice, VDR‐knockout, feeding without 1,25(OH)_2_D_3_

VDR, vitamin D receptor.

### Specimen collection and processing

After fed for 8, 16 and 24 weeks, the body weights of all mice were measured. Then, the mice were anaesthetized with intraperitoneal injection of 10% chloral hydrate in saline (Boster, Wuhan, China). The blood samples were drawn from retinal venous plexus. Heparin was added into the blood samples to prevent clotting. The samples were divided into two parts, one was used for the determination of T lymphocytes by flow cytometry, and the other was used to detect the expressions of inflammatory cytokines and biochemical indicators. Both kidneys in the mice were collected. Part of the kidney samples was used to detect the expressions of inflammatory cytokines by quantitative real‐time polymerase chain reaction (qRT‐PCR) and Western blotting. The other part of kidney samples was fixed in 10% neutral formalin, washed with PBS, dehydrated with gradient alcohol, processed with xylene and embedded in paraffin (Thermo Fisher Scientific Inc., Waltham, MA, USA). The embedded samples were cut into 5‐μm‐thick slices and were used for the observation of tissue morphology.

### Isolation of peripheral blood T lymphocytes

Tissue diluent (Invitrogen Inc., Carlsbad, CA, USA) was mixed with blood samples with a 1:1 ratio. Equal volume of lymphocyte separation medium (Invitrogen Inc.) was added carefully. The mixture was centrifuged at 626 g for 30 min. at 20°C in a Thermo centrifuge. After the centrifugation, there were four layers of visible cells in the centrifuge tube. The second layer that contained milky lymphocytes was collected and washed by adding 5 ml cell washing buffer (Invitrogen Inc.). The samples were then fully mixed and centrifuged for 10 min. at 2000 r.p.m. The supernatant was discarded, and the remaining precipitation was mononuclear cells. The cells were resuspended in 80 μl of immunomagnetic buffer solution and 20 μl of magnetic beads (Miltenyi biotec, Bergisch Gladbach, Germany) were added into the tube. The suspension was maintained at 4°C in the dark for 15 min. and centrifuged at 1000 r.p.m for 10 min. The supernatant was discarded, and 500 μl of buffer solution were added to resuspend the cells. The cells were then transferred into a magnetic separation column (Miltenyi biotec) which was washed twice with the buffer solution. At the end, the magnetic field was removed and the T lymphocytes were collected with 1 ml of RMPI‐1640 medium (Hyclone, Thermo Fisher Scientific Inc.).

### Flow cytometry

The collected T lymphocytes were diluted to 1 × 10^6^/ml. A volume of 100 μl cell suspension was collected in the Eppendorf tube for the measurement. Fluorescein isothiocyanate (FITC)‐labelled anti‐IgG1 antibody (IgG1‐FITC; Becton, Dickinson and Company, BD Bioscience, Sam Iose, CA, USA) and phycoerythrin (PE)‐labelled anti‐IgG antibody (IgG‐PE; Becton, Dickinson and Company) of 10 μl each were added into the negative control. In the testing samples, 10 μl of monoclonal antibody for CD4 (Becton, Dickinson and Company) or CD8 (Becton, Dickinson and Company) was added and incubated in the dark for 20 min. A volume of 100 μl haemolysin (Becton, Dickinson and Company) was subsequently added and incubated in the dark for 15 min. The mixture was centrifuged again, and the supernatant was discarded. The samples were then measured using flow cytometry (Becton, Dickinson and Company). The cells were sorted with a flow rate of 4000 cells per second. The number of analysed cells should not be less than 2000. The results were analysed with the CellQuest software. The changes in CD4^+^, CD4^+^/CD8^+^, DP, CD8^+^ and double‐negative (DN) cells were analysed with the CS2000 software.

### Detection of serum biochemical indexes

Blood urea nitrogen (BUN) was detected by continuously monitoring of the urease level according to the manufacturer's instruction of a reagent kit (Rongsheng biotech, Shanghai, China). In brief, 10 μl of samples was added into a test tube, and 10 μl of calibration solution was added into a control tube. The colorimetric values of the two tubes were immediately measured after mixing using a Thermo spectrophotometer. The absorbance of the mixture was measured at 30 sec. (A1) and 90 sec. (A2), and the serum level of BUN (mmol/l) was calculated as (A2 of sample − A1 of sample)/(A2 of calibration solution − A1 of calibration solution) × concentration of the calibration solution.

The serum creatinine (Cr) was detected by a picric acid method, according to the manual of the reagent kit (Rongsheng biotech). In brief, 100 μl of sample solution was added into a test tube, and 100 μl of calibration solution was added into a control tube. The tubes were incubated in a 37°C water bath for 30 sec., and the initial absorbance (A1) and the absorbance at 90 sec. (A2) were measured at the wavelength of 505 nm. The level of Cr (μmol/l) was calculated as (A2 of the sample − A1 of the sample)/(A2 of calibration solution − A1 of calibration solution) × concentration of the calibration solution.

### Enzyme‐linked immunosorbent assay (ELISA)

The ELISA measurements were conducted in strict accordance with the manual of the experimental kit (eBioscience, San Diego, CA, USA). In brief, the ELISA kit was equilibrated at room temperature for 20 min. before using it to prepare the experimental solutions. After the standard was dissolved, 100 μl of standard solution was added into the reaction plate to produce the standard curve. A volume of 100 μl sample solution was added into each well, and the plate was incubated at 37°C for 90 min. After washing the plate, 100 μl of promptly made working solution containing biotinylated antibodies was added into the wells and incubated at 37°C for 60 min. After the second washing, 100 μl of promptly made solution containing enzyme‐bound reactants were added and incubated in the dark at 37°C for 30 min. The plate was then washed consecutively for three times, and 100 μl of substrate solution were added and incubated in the dark at 37°C for 15 min. Finally, stop solution was quickly added into the plate to terminate the reactions, and within 3 min., the optical density (OD) values were measured at a wavelength of 450 nm using a multifunction plate reader (Synergy 2; Bio Tek Instruments, Inc., Winooski, VT, USA). The standard curve was drawn based on the measured OD values, and the plasma levels of interleukin‐4 (IL‐4), IL‐10, IL‐17 and interferon‐γ (INF‐γ) were calculated from the standard curve.

### Quantitative real‐time polymerase chain reaction (qRT‐PCR)

TRIzol (Invitrogen Inc.) was used to extract mice renal RNA. The RNA concentration and purity were measured with NanoDrop2000 (Thermo Fisher Scientific Inc.). According to the gene sequences published in the Genbank database, PCR primers were designed using the Primer5.0 software and were synthesized (GenePharma Co, Ltd., Shanghai, China). The primer sequences are shown in Table 2. The PCR was performed using the PRISM 7500 PCR System (Applied Biosystems, Foster City, CA, USA). The PCR system included 12.5 μl of SYBR premix ex Taq II (Takara Holdings Inc., Kyoto, Japan), 1 μl each of forward and reverse primers, 2 μl of DNA template and 8.5 μl of sterile water. The β‐actin was used as internal control. The PCR conditions were as follows: 94°C pre‐denaturation for 3 min.; 40 cycles of 94°C denaturation for 30 sec., 54°C annealing for 30 sec. and 72°C extension for 60 sec. The reliability of the PCR results was evaluated using the dissolution curve. The cycle threshold (*C*_t_) values were obtained to calculate the relative gene expressing using 2^−∆∆^ *C*_t_, where ∆*C*_t_ = *C*_t_ of target gene − *C*_t_ of internal control, and ∆∆*C*_t_ = ∆*C*_t_ (sample group) − ∆*C*_t_ (control group) 12.

**Table 2:** Primer sequences for the qRT‐PCR

Gene	Sequence
IL‐4	
Forward Primer	5′‐ GCTATTGATGGGTCTCACCC‐3′
Reverse Primer	5′‐CAGGACGTCAAGGTACAGGA‐3′
IL‐10	
Forward Primer	5′‐GGTTGCCAAGCCTTATCGGA‐3′
Reverse Primer	5′‐ACCTGCTCCACTGCCTTGCT‐3′
IL‐17	
Forward Primer	5′‐ CAAGACTGAACACCGACTAAG‐3′
Reverse Primer	5′‐ TCTCCAAAGGAAGCCTGA‐3′
INF‐γ	
Forward Primer	5′‐GAGGGATCCATGAAATATACAAGCTAT‐3′
Reverse Primer	5′‐GACGAATTCTTACGTTGATGCTCTCC‐3′
β‐Actin	
Forward Primer	5′‐ CCTAGAAGCATTTGCGGTGG‐3′
Reverse Primer	5′‐ GAGCTACGAGCTGCCTGACG ‐3′

qRT‐PCR, quantitative real‐time polymerase chain reaction; IL, interleukin; INF‐γ, interferon‐γ.

### Western blotting

Mouse renal proteins were extracted and the protein concentrations were measured by a BCA Kit (Boster). The extracted proteins were denatured by adding a buffer solution and boiled for 10 min. at 95°C. The proteins were then separated using 10% polyacrylamide gel electrophoresis (PAGE) (Boster). During PAGE, each well was added with 40 μg of samples, and the electrophoresis voltages were 80 V in concentrated gels and then 120 V in separation gels. The transferring membrane was polyvinylidene fluoride (PVDF). Wet transfer was used with a transferring voltage of 100 mV. The operation time of transfer was 60 min. and sealed at room temperature and in 5% bovine serum albumin (BSA) for 1 hr. The primary antibodies were all used at a 1:1000 dilution, including phosphorylated extracellular signal‐regulated kinase‐1/2 (p‐ERK1/2, ab50011; Abcam, Cambridge, UK), total ERK1/2 (t‐ERK1/2, ab54230; Abcam, UK) and β‐actin (ab6276; Abcam, UK). During primary antibody staining, the samples were incubated overnight at 4°C. After the staining, the samples were rinsed three times by a Tris‐buffered saline and Tween‐20 (TBST) solutions, with each rinse taking 5 min. Secondary antibodies were then added and incubated at room temperature for 1 hr. The samples were rinsed three times again by TBST, with each rinse taking 5 min. The samples were then developed using a chemical luminescence reagent, and the grey values of the target bands were analysed by the ImageJ software using β‐actin as the internal control.

### Haematoxylin–eosin (HE) staining

The pathological morphology of sample tissues was observed by HE staining, which was beneficial to increase the colour difference among different parts of the tissue and cell structures. Paraffin sections of renal tissues were dewaxed using xylene (Boster) and hydrated using gradient alcohol (Boster). After staining with haematoxylin (Boster) for 10 min., the slides were washed with water. The slides were subsequently differentiated with a hydrogen chloride and ethanol solution (Boster) for 30 sec., and stained in blue using ammonia (Boster) for 30 sec. Finally, the slides were washed twice with water, counterstained by eosin for 3 min., dehydrated with alcohol and made transparent using xylene (Boster). The slides were mounted in neutral resin and placed under a microscope (Cx31, Olympus, Japan) to observe the pathology of renal tissues.

### Immunofluorescence assay

Immunoglobulins IgG, IgA, IgM, C3 and C1q were labelled with FITC. Renal biopsy slides were dewaxed and processed in 3% catalase for 10 min. After subsequent treatment by pepsin for 10 min., the slides were incubated in Gibco calf serum at 37°C for 1 hr. The slides then underwent immunofluorescence staining by adding corresponding fluorescent antibodies including IgG (1:100 dilution), IgA (1:100 dilution), IgM (1:80 dilution), C3 (1:50 dilution) and C1q (1:50 dilution). All antibodies and reagents were purchased from DAKO Company (Glostrup, Denmark). During antibody staining, the slides were placed in a wet box and incubated at 37°C for 45 min. After the staining, the slides were washed with TBST and then dried at room temperature. The slides were then mounted by adding a glycerol buffer DAKO Company. In order to detect the intensity of immune complex precipitation, the slides were imaged under a fluorescent microscope.

### Statistical analysis

SPSS18.0 statistical software was used for statistical analyses. Measurement data were presented as mean ± standard deviations. Differences of measurement data of the normal distributions among multiple groups were analysed using one‐way analysis of variance (anova), and the differences between two groups were compared using LSD *t*‐test. *P* < 0.05 indicated the difference was statistically significant.

## Results

### Comparisons of general conditions of mice in each group

**Figure 1:**
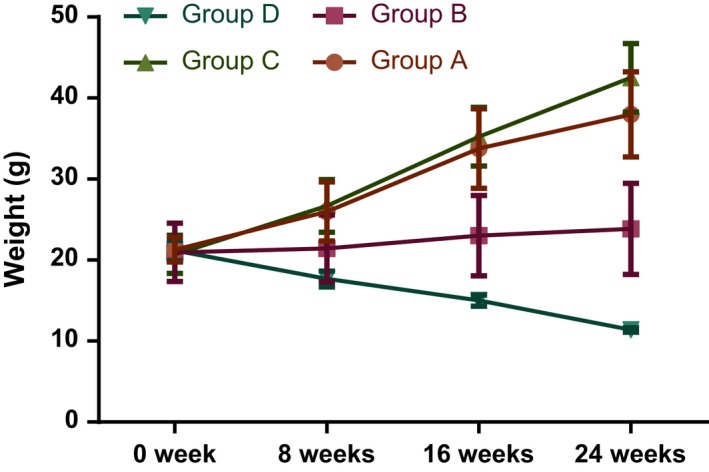
Comparison of body weight changes in mice in each group.

The weight of mice in the 1,25(OH)_2_D_3_ group was elevated with the increase in time. The general conditions of mice were significantly improved, and the severity of skin ulcers and urinary system infections were significantly reduced. In the VDR‐knock‐in + 1,25(OH)_2_D_3_ group, the weight of mice was also raised with the increase in time, but the difference was not significantly different. In addition, the severity of loosening hair, skin ulcers and urinary system infection was not significantly improved. In the control group, the weight of mice was increased along with the time, and there was no skin ulcer or urinary system infection. In the VDR‐knockout group, the weight of mice was decreased with the increase in time, and the fur became loose. In addition, skin ulcers and urinary tract infections occurred (Figure 1).

### Comparisons of T lymphocyte parameters of mice in each group

Compared with normal mice, the levels of CD4^+^CD8^−^ single‐positive (CD4^+^SP), CD4^+^/CD8^+^ and CD4^+^CD8^+^ DP T lymphocytes were decreased in SLE mice, while the levels of CD8^+^CD4^−^ single‐positive (CD8^+^SP) and CD_4_^−^CD_8_^−^ DN T lymphocytes were increased in SLE mice (all *P* < 0.05). In the VDR‐knock‐in + 1,25(OH)_2_D_3_ group, the levels of CD4^+^SP, CD4^+^/CD8^+^ and DP T lymphocytes were elevated, while the levels of CD8^+^SP and DN T lymphocytes were reduced along with the time (all *P* < 0.05). At 24 weeks after the treatment, there were no significant differences between the 1,25(OH)_2_D_3_ and the control groups. In the VDR‐knock‐in + 1,25(OH)_2_D_3_ and the control groups, the T lymphocyte parameters had no significant changes along with the time (all *P* > 0.05). However, in the VDR‐knockout group, the levels of CD_4_^+^SP, CD4^+^/CD8^+^ and DP T lymphocytes were decreased, while the levels of CD_8_^+^SP and DN T lymphocytes were increased along with time (all *P* < 0.05) (Table 3).

**Table 3:** Comparisons of different subsets of T lymphocytes in each group (%)

	CD_4_^+^SP (%)	CD_8_^+^SP (%)	CD_4_^+^/CD_8_^+^	DP (%)	DN (%)
1,25(OH)_2_D_3_ group					
8 weeks	30.09 ± 3.92§	18.06 ± 3.53§	2.72 ± 0.59§	0.64 ± 0.13§	3.76 ± 0.69§
16 weeks	36.58 ± 4.28*, ‡, §	14.03 ± 3.28*, ‡, §	3.68 ± 0.74*, ‡, §	1.21 ± 0.06*, ‡, §	2.68 ± 0.35*, ‡, §
24 weeks	49.85 ± 5.16*, †, ‡	10.81 ± 2.96*, †, ‡	4.79 ± 1.64*, †, ‡	2.54 ± 0.12*, †, ‡	1.38 ± 0.24*, †, ‡
VDR‐knock‐in + 1,25(OH)_2_D_3_ group					
8 weeks	29.64 ± 6.30§	17.23 ± 3.65§	2.37 ± 0.81§	0.60 ± 0.23§	3.62 ± 1.00§
16 weeks	29.83 ± 4.90§	18.15 ± 2.95§	2.46 ± 0.29§	0.68 ± 0.20§	3.73 ± 0.72§
24 weeks	30.50 ± 9.00§	18.36 ± 5.10§	2.62 ± 1.11§	0.71 ± 0.40§	3.79 ± 0.91§
Control group					
8 weeks	53.12 ± 8.2	10.35 ± 2.32	5.43 ± 1.69	2.70 ± 0.51	1.03 ± 0.24
16 weeks	51.14 ± 9.7	10.29 ± 1.39	5.09 ± 1.31	2.65 ± 0.41	1.09 ± 0.38
24 weeks	50.98 ± 10.2	10.96 ± 4.1	5.19 ± 1.92	2.59 ± 0.26	0.97 ± 0.24
VDR‐knockout group					
8 weeks	48.56 ± 5.32	11.04 ± 2.68	5.02 ± 0.41	2.69 ± 0.59	1.22 ± 0.34
16 weeks	39.95 ± 4.85*, §	19.65 ± 3.74*, §	3.78 ± 0.39*, §	1.16 ± 0.31*, §	2.93 ± 0.60*, §
24 weeks	31.92 ± 3.62*, †, §	23.65 ± 5.30*, †, §	1.75 ± 0.24†, §	0.65 ± 0.14*, †, §	3.49 ± 0.59*, †, §

*indicating *P* < 0.05 as compared to the values at 8 weeks; ^†^indicating *P* < 0.05 as compared to the values at 16 weeks; ^‡^indicating *P* < 0.05 as compared to the VDR‐knock‐in + 1,25(OH)_2_D_3_ group; ^§^indicating *P* < 0.05 as compared to the control group; SP, single‐positive; DP, double‐positive; DN, double‐negative.

### Comparisons of blood biochemical indexes of mice in each group

Compared with the normal mice, the levels of BUN and Cr were significantly increased in SLE mice (both *P* < 0.05). The levels of BUN and Cr were significantly reduced along with the time in the 1,25(OH)_2_D_3_ group. At 24 weeks after the treatment, there were no significant changes in levels of BUN and Cr between the 1,25(OH)_2_D_3_ and the control groups. In the VDR‐knock‐in + 1,25(OH)_2_D_3_ and the control groups, the levels of BUN and Cr had no significant changes along with the time (all *P* > 0.05). However, in the VDR‐knockout group, the levels of BUN and Cr were increased along with time (all *P* < 0.05) (Table 4).

**Table 4:** Comparisons of blood biochemical indexes in each group

	BUN (μmol/l)	Cr (μmol/l)
1,25(OH)_2_D_3_ group		
8 weeks	23.01 ± 3.65§	88.81 ± 8.65§
16 weeks	16.53 ± 2.32*, ‡, §	76.31 ± 7.14*, ‡, §
24 weeks	9.36 ± 1.29*, †, ‡	49.12 ± 5.18*, †, ‡
VDR‐knock‐in + 1,25(OH)_2_D_3_ group		
8 weeks	22.03 ± 2.36§	86.71 ± 7.26§
16 weeks	21.98 ± 1.27§	85.89 ± 5.62§
24 weeks	20.52 ± 2.36§	83.32 ± 9.20§
Control group		
8 weeks	9.15 ± 1.02	50.15 ± 4.21
16 weeks	8.95 ± 0.96	46.89 ± 3.09
24 weeks	10.01 ± 2.63	49.31 ± 3.21
VDR‐knockout group		
8 weeks	9.29 ± 0.29	47.65 ± 4.31
16 weeks	14.57 ± 1.21*, §	58.25 ± 5.26*, §
24 weeks	19.32 ± 1.32*, †, §	86.78 ± 6.13*, †, §

*indicating *P* < 0.05 as compared to the values at 8 weeks; ^†^indicating *P* < 0.05 as compared to the values at 16 weeks; ^‡^indicating *P* < 0.05 as compared to the VDR‐knock‐in + 1,25(OH)_2_D_3_ group; ^§^indicating *P* < 0.05 as compared to the control group; BUN, blood urea nitrogen; Cr, creatinine.

### Comparisons of expressions of IL‐4, IL‐10, IL‐17 and INF‐γ in each group

**Figure 2:**
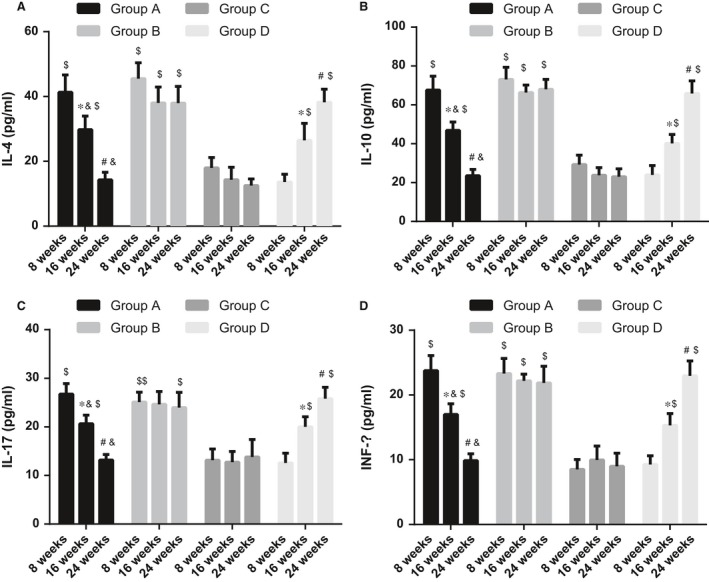
Comparisons of expressions of IL‐4, IL‐10, IL‐17 and INF‐γ in each group. (**A**), IL‐4 protein expression at different time‐points in each group; (**B**), IL‐10 protein expression at different time‐points in each group; (**C**), IL‐17 protein expression at different time‐points in each group; (**D**), interferon‐γ protein expression at different time‐points in each group; *indicating *P* < 0.05 as compared to the values at 8 weeks; ^#^indicating *P* < 0.05 as compared to the values at 16 weeks; ^&^indicating *P* < 0.05 as compared to the VDR‐knock‐in + 1,25(OH)_2_D_3_ group; ^$^indicating *P* < 0.05 as compared to the control group; IL, interleukin; INF‐γ, interferon gamma.

The expressions of inflammatory cytokines were measured by ELISA. Compared with normal mice, the expressions of IL‐4, IL‐10, IL‐17 and INF‐γ were significantly increased in SLE mice (all *P* < 0.05). In the VDR‐knock‐in + 1,25(OH)_2_D_3_ group, the expressions of IL‐4, IL‐10, IL‐17 and INF‐γ were significantly reduced along with the time (all *P* < 0.05). At 24 weeks after the treatment, there were no significant changes in expressions of IL‐4, IL‐10, IL‐17 and INF‐γ between the 1,25(OH)_2_D_3_ and the control groups. In the VDR‐knock‐in + 1,25(OH)_2_D_3_ and the control groups, the expressions of IL‐4, IL‐10, IL‐17 and INF‐γ had no significant changes along with the time (all *P* > 0.05). However, in the VDR‐knockout group, the expressions of IL‐4, IL‐10, IL‐17 and INF‐γ were increased along with time (all *P* < 0.05) (Figure 2).

### Comparisons of expressions of IL‐4, IL‐10, IL‐17 and INF‐γ mRNA in each group

**Figure 3:**
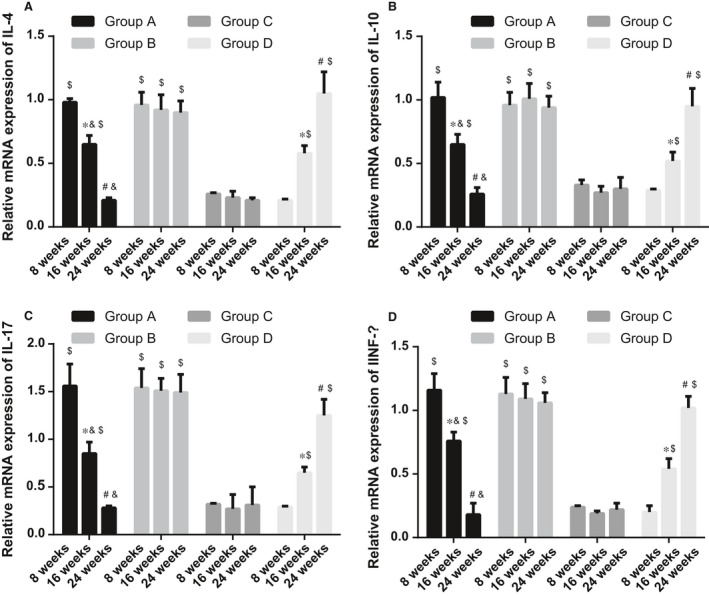
Expressions of IL‐4, IL‐10, IL‐17 and INF *‐*γ mRNA in each group. (**A**), *IL‐4* mRNA expression at different time‐points in each group; (**B**), *IL‐10* mRNA expression at different time‐points in each group; (**C**), *IL‐17* mRNA expression at different time‐points in each group; (**D**), *INF‐*γ mRNA expression at different time‐points in each group; *indicating *P* < 0.05 as compared to the values at 8 weeks; ^#^indicating *P* < 0.05 as compared to the values at 16 weeks; ^&^indicating *P* < 0.05 as compared to the VDR‐knock‐in + 1,25(OH)2D3 group; ^$^indicating *P* < 0.05 as compared to the control group; IL, interleukin; INF‐γ, interferon gamma.

The mRNA expressions of inflammatory cytokines were measured using qRT‐PCR. Compared with normal mice, the expressions of *IL‐4*,* IL‐10*,* IL‐17* and *INF‐*γ mRNA were significantly elevated in SLE mice (all *P* < 0.05). In the 1,25(OH)_2_D_3_ group, the expressions of *IL‐4*,* I L‐10*,* IL‐17* and *INF‐*γ mRNA were decreased in SLE mice along with the time (all *P* < 0.05). At 24 weeks after the treatment, there were no significant changes in expressions of *IL‐4, IL‐10, IL‐17 and INF‐*γ mRNA between the normal and SLE mice. In the VDR‐knock‐in + 1,25(OH)_2_D_3_ and the control groups, the expressions of *IL‐4, IL‐10, IL‐17 and INF‐*γ mRNA had no significant changes along with the time (all *P* > 0.05). However, in the VDR‐knockout group, the expressions of *IL‐4, IL‐10, IL‐17 and INF‐*γ mRNA were gradually increased along with the time (all *P* < 0.05) (Figure 3).

### Comparisons of ERK1/2 expression and phosphorylation in each group

**Figure 4:**
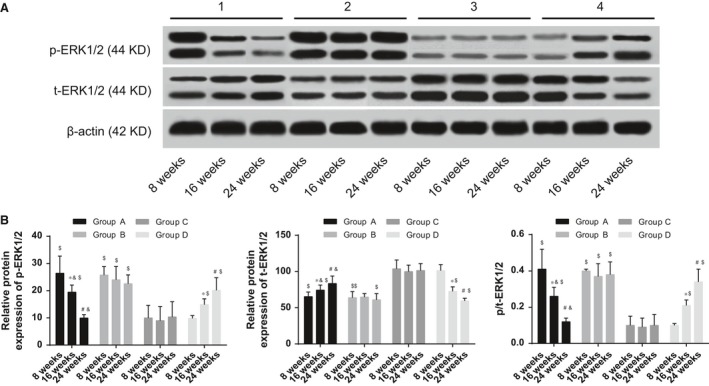
Comparisons of ERK1/2 expression and phosphorylation in each group. (**A**), expressions of p‐ERK1/2 and t‐ERK1/2 at different time‐points in each group; (**B**), grey band analysis of p‐ERK1/2 in t‐ERK1/2 at different time‐points in each group; *indicating *P* < 0.05 as compared to the values at 8 weeks; ^#^indicating *P* < 0.05 as compared to the values at 16 weeks; ^&^indicating *P* < 0.05 as compared to the VDR‐knock‐in + 1,25(OH)2D3 group; ^$^indicating *P* < 0.05 as compared to the control group; ERK, extracellular regulated protein kinase.

Compared with normal mice, the p‐ERK1/2 expression was increased, while the t‐ERK1/2 expression was decreased in SLE mice, and the ratio of p/t‐ERK1/2 was significantly increased (all *P* < 0.05). In the 1,25(OH)_2_D_3_ group, the p‐ERK1/2 expression was reduced, the t‐ERK1/2 expression was elevated, and the ratio of p/t‐ERK1/2 was decreased along with the time (all *P* < 0.05). At 24 weeks after the treatment, there were no significant changes in p‐ERK1/2 expression between the 1,25(OH)_2_D_3_ and the control groups. In the VDR‐knock‐in + 1,25(OH)_2_D_3_ and the control groups, the expressions of p‐ERK1/2 and t‐ERK1/2 as well as the ratio of p/t‐ERK1/2 had no significant changes along with the time (all *P* > 0.05). However, in the VDR‐knockout group, the p‐ERK1/2 expression was gradually increased, the t‐ERK1/2 expression was gradually decreased, and the ratio of p/t‐ERK1/2 was elevated along with the time (all *P* < 0.05) (Figure 4).

### Comparisons of histopathological observation of mice in each group

**Figure 5:**
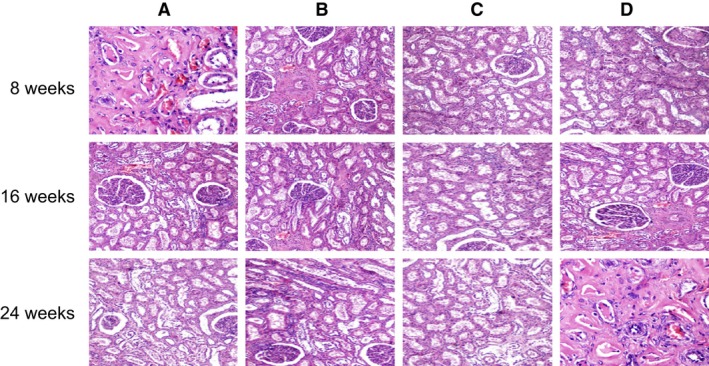
Histopathological observation of mice tissues in each group (400×). The arrows indicate the infiltration of inflammatory cells; (**A**), MRL‐LPr/LPr mice, feeding with 1,25(OH)_2_D_3;_ (**B**), MRL‐LPr/LPr mice, VDR‐knock‐in and feeding with 1,25(OH)_2_D_3;_ (**C**), normal mice, feeding without 1,25(OH)_2_D_3;_ (**D**), normal mice, VDR‐knockout and feeding without 1,25(OH)_2_D_3_.

In normal mice, the morphology of renal tissues showed no histopathological changes. In SLE mice, severe renal injuries were observed, the glomerular volume and the number of cells were both increased, the glomerular lobules formed with thickening of the basement membrane, inflammatory cell infiltration was seen in glomerular and interstitial tissues, and mononuclear cell infiltration was seen in glomerular capillaries. In the 1,25(OH)_2_D_3_ group, renal injuries were improved, and inflammatory cell infiltration was reduced along with the time (both *P* < 0.05). At 24 weeks after the treatment, renal injuries of SLE mice were improved, and there were no significant changes in inflammatory cell infiltration between the normal and SLE mice. In the VDR‐knock‐in + 1,25(OH)_2_D_3_ and the control groups, renal injuries and inflammatory cell infiltration had no significant changes along with the time (all *P* > 0.05). However, in the VDR‐knockout group, the degree of renal injuries and inflammatory cell infiltration were increased along with the time, and SLE symptoms gradually occurred in the mice of VDR‐knockout group (all *P* < 0.05) (Figure 5).

### Comparisons of histopathological changes in mice tissues in each group

**Figure 6:**
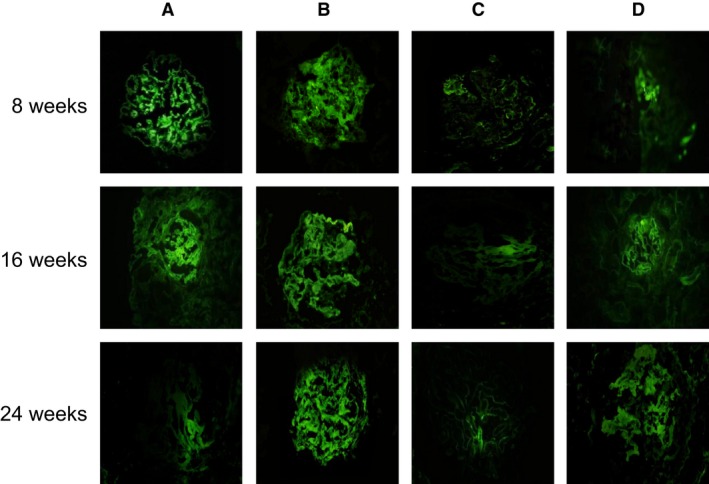
Histopathological changes in mice tissues in each group observed by the immunofluorescence assay (100×). (**A**), MRL‐LPr/LPr mice, feeding with 1,25(OH)_2_D_3;_ (**B**), MRL‐LPr/LPr mice, VDR‐knock‐in and feeding with 1,25(OH)_2_D_3;_ (**C**), normal mice, feeding without 1,25(OH)_2_D_3;_ (**D**), normal mice, VDR‐knockout and feeding without 1,25(OH)_2_D_3_.

Compared with the normal mice, the levels of immunoglobulin and complement deposition increased in SLE mice, and the immunofluorescence staining of the renal tissues showed positive signals for IgG, IgA, IgM, C_3_ and C_1q_ at the same time, which is a characteristic immune pathological change for lupus nephritis. In addition, microscopically visible immune complex deposition in a variety of different sizes was seen in the glomerular endothelium, epithelium, mesangial and tubular basement membrane. In the 1,25(OH)_2_D_3_ group, the levels of immunoglobulin and complement deposition were decreased gradually along with the time (all *P* < 0.05). At 24 weeks after the treatment, there were no significant changes in the levels of immunoglobulin and complement deposition between the normal and SLE mice. In the VDR‐knock‐in + 1,25(OH)_2_D_3_ and the control groups, the level of complement deposition had no significant changes along with the time (all *P* > 0.05). However, in the VDR‐knockout group, the level of complement deposition was gradually increased along with the time, and SLE symptoms gradually occurred in the mice of VDR‐knockout group (all *P* < 0.05) (Figure 6).

## Discussion

The purpose of this study is to elucidate the effects of 1,25(OH)_2_D_3_ and *VDR* on peripheral blood T lymphocytes in SLE. The results showed that 1,25(OH)_2_D_3_ and *VDR* were associated with the occurrence of SLE, suggesting that they may become new targets for the treatment of SLE.

SLE is a systemic autoimmune disease with clinical heterogeneity, and some specific genes can increase the susceptibility to SLE 13. The VD deficiency may play an important role in the occurrence of some autocrine diseases 14. SLE is usually accompanied by abnormal immune responses including inadequate regulation of B cells by T cells and excessive secretion of autoimmune antibodies, and the VD of many SLE patients is deficient, with the level of 1,25(OH)_2_D_3_ negatively related to the prognosis of SLE 15.

In this study, feeding with 1,25(OH)_2_D_3_ significantly improved the health of SLE mice, while the health conditions of normal mice which are insufficient in VDR deteriorate gradually along with the time. 1,25(OH)_2_D_3_, the active form of vitamin D, mainly functions on calcium homeostasis and bone metabolism. It is located in 12q of the chromosome and includes more than 470 sites of single nucleotide polymorphism (SNP), with some SNPs upregulating the level of 1,25(OH)_2_D_3_ 16. The risk of VD deficiency is higher in SLE patients than in the general population, and VD deficiency can lead to B‐cell hyperactivation and production of autoimmune antibodies in genetically susceptible populations, and *VDR* is expressed in all types of cells of the immune system 17. Mechanisms of transmembrane signal transduction can cause rapid biological effects of 1,25(OH)_2_D_3_, which are mainly activated by 1,25(OH)_2_D_3_ inducing the phosphorylation of some signal transduction molecules, such as protein kinase C (PKC) and ERK1/2; 1,25(OH)_2_D_3_ can modulate the PKC activity, thus activating the Raf in the upstream of the ERK1/2 pathway, which lead to the phosphorylation of ERK1/2 18, 19, 20. Boyan *et al*. 21 found that 1,25(OH)_2_D_3_ can rapidly induce the phosphorylation of PKC and ERK1/2 in growth zone cells, and the induction of 1,25(OH)_2_D_3_ on ERK1/2 phosphorylation is mediated by PKC. According to Deeb *et al*., 22 rapid actions of 1,25(OH)_2_D_3_ involve 1,25(OH)_2_D_3_ binding with the cytosolic and membrane VDR to activate PKC through phosphorylation, thus activating ERK pathway through Ras‐Raf‐MEK‐ERK cascade. A previous study demonstrates that 1,25(OH)_2_D_3_ interferes the ERK signalling pathway to inhibit tumour cell growth 23. It has been shown that during the treatment of SLE patients, the increase in 1,25‐(OH)_2_D_3_ dosage inhibited the activity of SLE 24, similar to the results from this study. In addition, a close association has been found between *VDR* polymorphisms and SLE severity 10. In this study, the mice with *VDR* deficiency showed deteriorating health conditions, indicating that the VDR deficiency may increase the risk of SLE.

In this study, it was found that as compared to normal mice, the levels of CD_4_^+^SP, CD4^+^/CD8^+^ and DP in SLE mice were decreased, while the number of CD_8_^+^SP and DN cells was increased, indicating certain immune abnormalities. The ability of 1,25(OH)_2_D_3_ to suppress inflammation has been linked to its capacity to regulate DC and T‐cell functions 25. Autoreactive T lymphocyte activation can lead to abnormal numbers of CD4^+^ and CD8^+^ T cells, and these changes can further induce SLE; in SLE patients, the number of CD4^+^ T cells was decreased, the number of CD8^+^ T cells was increased, and the ratio of CD4^+^/CD8^+^ T cells was continued to decline 26.

In this study, it was also shown that as compared with normal mice, the protein and mRNA expressions of IL‐4, IL‐10, IL‐17 and INF‐γ in SLE mice were significantly increased. Mature CD4^+^ T cells can be divided into two distinct subgroups (Th1 and Th2) based on different expressions of cell factors 27. In SLE patients, the serum levels of Th1 and Th2 cytokines increased, suggesting that they may participate in the inflammation and tissue damage of SLE 28. INF‐γ is produced by Th1 cells, while IL‐4 and IL‐10 are produced by Th2 cells, and IL‐17 is mainly secreted by Th1 29. Secretion of IL‐17 promotes inflammation, and its expression in T cells is associated with the activity of SLE. Due to the positive association between IL‐17 and INF‐γ, the increase in INF‐γ in the serum of SLE patients reasonably upregulated the IL‐17 expression and it has been shown that IL‐17 plays an important role in various autocrine diseases, including SLE and rheumatoid arthritis 30, 31.

In summary, this study found that 1,25(OH)_2_D_3_ and *VDR* participated in the occurrence of SLE by regulating cytokine secretion in different subsets of T cells, and they may become new drug targets during the treatment of SLE. However, there are still limitations in this research, and the exact mechanisms of 1,25(OH)_2_D_3_ and *VDR* remain elusive, which require further studies.

## Competing interests

None.

## References

[1] D'Cruz DP , Khamashta MA , Hughes GR . Systemic lupus erythematosus. Lancet. 2007; 369: 587–96. 17307106 10.1016/S0140-6736(07)60279-7

[2] Ribi C , Trendelenburg M , Gayet‐Ageron A , *et al* The Swiss systemic lupus erythematosus Cohort Study (SSCS) – cross‐sectional analysis of clinical characteristics and treatments across different medical disciplines in Switzerland. Swiss Med Wkly. 2014; 144: w13990. 25115978 10.4414/smw.2014.13990

[3] Li X , Wang Y . Systemic lupus erythematosus with acute inflammatory demyelinatingpolyneuropathy: a case report and review of the literature. J Clin Med Res. 2016; 8: 555–9. 27298667 10.14740/jocmr2550w PMC4894028

[4] Son M , Kim SJ , Diamond B . SLE‐associated risk factors affect DC function. Immunol Rev. 2016; 269: 100–17. 26683148 10.1111/imr.12348 PMC4685736

[5] Deng Y , Tsao BP . Genetic susceptibility to systemic lupus erythematosus in the genomic era. Nat Rev Rheumatol. 2010; 6: 683–92. 21060334 10.1038/nrrheum.2010.176 PMC3135416

[6] Handono K , Marisa D , Kalim H . Association between the low levels of vitamin D and Treg function in systemic lupus erythematosus patients. Acta Med Indones. 2013; 45: 26–31. 23585405

[7] Ramos‐Martinez E , Villasenor‐Cardoso MI , Lopez‐Vancell MR , *et al* Effect of 1,25(OH)2D3 on BALB/c mice infected with Leishmania mexicana. Exp Parasitol. 2013; 134: 413–21. 23707346 10.1016/j.exppara.2013.05.009

[8] Wahono CS , Rusmini H , Soelistyoningsih D , *et al* Effects of 1,25(OH)2D3 in immune response regulation of systemic lupus erithematosus (SLE) patient with hypovitamin D. Int J Clin Exp Med. 2014; 7: 22–31. 24482685 PMC3902237

[9] Haussler MR , Jurutka PW , Mizwicki M , *et al* Vitamin D receptor (VDR)‐mediated actions of 1alpha,25(OH)(2)vitamin D(3): genomic and non‐genomic mechanisms. Best Pract Res Clin Endocrinol Metab. 2011; 25: 543–59. 21872797 10.1016/j.beem.2011.05.010

[10] Carvalho C , Marinho A , Leal B , *et al* Association between vitamin D receptor (VDR) gene polymorphisms and systemic lupus erythematosus in Portuguese patients. Lupus. 2015; 24: 846–53. 25661837 10.1177/0961203314566636

[11] Orlans FB . Ethical decision making about animal experiments. Ethics Behav. 1997; 7: 163–71. 11655130 10.1207/s15327019eb0702_7

[12] Tuo YL , Li XM , Luo J . Long noncoding RNA UCA1 modulates breast cancer cell growth and apoptosis through decreasing tumor suppressive miR‐143. Eur Rev Med Pharmacol Sci. 2015; 19: 3403–11. 26439035

[13] Vincent FB , Morand EF , Schneider P , *et al* The BAFF/APRIL system in SLE pathogenesis. Nat Rev Rheumatol. 2014; 10: 365–73. 24614588 10.1038/nrrheum.2014.33

[14] Szodoray P , Tarr T , Bazso A , *et al* The immunopathological role of vitamin D in patients with SLE: data from a single centre registry in Hungary. Scand J Rheumatol. 2011; 40: 122–6. 20977384 10.3109/03009742.2010.507220

[15] Mok CC , Birmingham DJ , Leung HW , *et al* Vitamin D levels in Chinese patients with systemic lupus erythematosus: relationship with disease activity, vascular risk factors and atherosclerosis. Rheumatology (Oxford). 2012; 51: 644–52. 21719424 10.1093/rheumatology/ker212

[16] Mostowska A , Lianeri M , Wudarski M , *et al* Vitamin D receptor gene BsmI, FokI, ApaI and TaqI polymorphisms and the risk of systemic lupus erythematosus. Mol Biol Rep. 2013; 40: 803–10. 23065277 10.1007/s11033-012-2118-6 PMC3538008

[17] Singh A , Kamen DL . Potential benefits of vitamin D for patients with systemic lupus erythematosus. Dermatoendocrinol. 2012; 4: 146–51. 22928070 10.4161/derm.20443 PMC3427193

[18] Boyan BD , Jennings EG , Wang L , *et al* Mechanisms regulating differential activation of membrane‐mediated signaling by 1alpha,25(oh)2d3 and 24r,25(oh)2d3. J Steroid Biochem Mol Biol. 2004; 89–90: 309–15. 10.1016/j.jsbmb.2004.03.027 15225791

[19] Capiati DA , Vazquez G , Tellez Inon MT , *et al* Role of protein kinase c in 1,25(oh)(2)‐vitamin d(3) modulation of intracellular calcium during development of skeletal muscle cells in culture. J Cell Biochem. 2000; 77: 200–12. 10723087 10.1002/(sici)1097-4644(20000501)77:2<200::aid-jcb4>3.0.co;2-5

[20] Hmama Z , Nandan D , Sly L , *et al* 1alpha,25‐dihydroxyvitamin d(3)‐induced myeloid cell differentiation is regulated by a vitamin d receptor‐phosphatidylinositol 3‐kinase signaling complex. J Exp Med. 1999; 190: 1583–94. 10587349 10.1084/jem.190.11.1583 PMC2195730

[21] Boyan BD , Posner GH , Greising DM , *et al* Hybrid structural analogues of 1,25‐(oh)2d3 regulate chondrocyte proliferation and proteoglycan production as well as protein kinase c through a nongenomic pathway. J Cell Biochem. 1997; 66: 457–70. 9282324

[22] Deeb KK , Trump DL , Johnson CS . Vitamin d signalling pathways in cancer: potential for anticancer therapeutics. Nat Rev Cancer. 2007; 7: 684–700. 17721433 10.1038/nrc2196

[23] Chattopadhyay N , MacLeod RJ , Tfelt‐Hansen J , *et al* 1alpha,25(oh)2‐vitamin d3 inhibits hgf synthesis and secretion from mg‐63 human osteosarcoma cells. Am J Physiol Endocrinol Metab. 2003; 284: E219–27. 12388161 10.1152/ajpendo.00247.2002

[24] Chaiamnuay S , Chailurkit LO , Narongroeknawin P , *et al* Current daily glucocorticoid use and serum creatinine levels are associated with lower 25(OH) vitamin D levels in Thai patients with systemic lupus erythematosus. J Clin Rheumatol. 2013; 19: 121–5. 23519176 10.1097/RHU.0b013e318289bd16

[25] Ehrchen J , Helming L , Varga G , *et al* Vitamin D receptor signaling contributes to susceptibility to infection with Leishmania major. FASEB J. 2007; 21: 3208–18. 17551101 10.1096/fj.06-7261com

[26] Shah D , Kiran R , Wanchu A , *et al* Relationship between T lymphocyte subsets and cortisol in systemic lupus erythematosus. Kathmandu Univ Med J (KUMJ). 2009; 7: 213–9. 20071865 10.3126/kumj.v7i3.2726

[27] Elser B , Lohoff M , Kock S , *et al* IFN‐gamma represses IL‐4 expression *via* IRF‐1 and IRF‐2. Immunity. 2002; 17: 703–12. 12479817 10.1016/s1074-7613(02)00471-5

[28] Henriques A , Ines L , Couto M , *et al* Frequency and functional activity of Th17, Tc17 and other T‐cell subsets in Systemic Lupus Erythematosus. Cell Immunol. 2010; 264: 97–103. 20553755 10.1016/j.cellimm.2010.05.004

[29] Yu Y , Ren XR , Wen F , *et al* T‐helper‐associated cytokines expression by peripheral blood mononuclear cells in patients with polypoidal choroidal vasculopathy and age‐related macular degeneration. BMC Ophthalmol. 2016; 16: 80. 27266510 10.1186/s12886-016-0251-z PMC4895798

[30] Dolff S , Quandt D , Wilde B , *et al* Increased expression of costimulatory markers CD134 and CD80 on interleukin‐17 producing T cells in patients with systemic lupus erythematosus. Arthritis Res Ther. 2010; 12: R150. 20653937 10.1186/ar3100 PMC2945048

[31] Shen H , Goodall JC , Hill Gaston JS . Frequency and phenotype of peripheral blood Th17 cells in ankylosing spondylitis and rheumatoid arthritis. Arthritis Rheum. 2009; 60: 1647–56. 19479869 10.1002/art.24568

